# Phytocystatins: Defense Proteins against Phytophagous Insects and Acari

**DOI:** 10.3390/ijms17101747

**Published:** 2016-10-20

**Authors:** Manuel Martinez, Maria Estrella Santamaria, Mercedes Diaz-Mendoza, Ana Arnaiz, Laura Carrillo, Felix Ortego, Isabel Diaz

**Affiliations:** 1 Centro de Biotecnologia y Genomica de Plantas, Universidad Politecnica de Madrid (UPM), Instituto Nacional de Investigacion y Tecnología Agraria y Alimentaria (INIA), Campus Montegancedo, Pozuelo de Alarcon, Madrid 28223, Spain; m.martinez@upm.es (M.M.); me.santamaria@upm.es (M.E.S.); mercedes.diaz.mendoza@upm.es (M.D.-M.); ana.arnaiz.alonso@alumnos.upm.es (A.A.); laura.carrillo@upm.es (L.C.); 2 Departamento de Biologia Medioambiental, Centro de Investigaciones Biologicas, CSIC, Ramiro de Maeztu, 9, Madrid 28040, Spain; ortego@cib.csic.es

**Keywords:** phytocystatin, insect, acari, digestive proteases, plant-phytophagous interactions

## Abstract

This review deals with phytocystatins, focussing on their potential role as defence proteins against phytophagous arthropods. Information about the evolutionary, molecular and biochemical features and inhibitory properties of phytocystatins are presented. Cystatin ability to inhibit heterologous cysteine protease activities is commented on as well as some approaches of tailoring cystatin specificity to enhance their defence function towards pests. A general landscape on the digestive proteases of phytophagous insects and acari and the remarkable plasticity of their digestive physiology after feeding on cystatins are highlighted. Biotechnological approaches to produce recombinant cystatins to be added to artificial diets or to be sprayed as insecticide–acaricide compounds and the of use cystatins as transgenes are discussed. Multiple examples and applications are included to end with some conclusions and future perspectives.

## 1. Phytocystatin Features

Peptidase inhibitory proteins are a complex group of molecules involved in the regulation of the protein degradation caused by peptidases. The MEROPS database (http://merops.sanger.ac.uk) is an integrated source of information that classifies peptidases and their inhibitors into families and clans [1]. Members of 21 of the 78 families recognized in the current MEROPS 10.0 version have been identified in plants [2]. From that, some of the most abundant peptidase inhibitors are the phytocystatins (PhyCys), which belong to the ubiquitous family of the cystatins (MEROPS identifier I25) [3]. Unique structural features and phylogenetic inferences suggest a specific evolution for PhyCys and support their inclusion in a specific plant cystatin family [4]. PhyCys are present in all land plants and in the Chlorophyceae algae. Their number progressively increased on evolution from one member in algae species to three in the pseudofern *Selaginella moellendorffii* and five in the moss *Physcomitrella patens*. In angiosperms, a broad range of members is found, with extensive species/clade specific duplications leading to 26 members in the grass species *Brachypodium distachion* [2]. Most PhyCys are small proteins with a molecular mass in the 12–16 kDa range and are inhibitors of the cysteine proteases (CysProt) from the C1A papain-like family. In land plants, some members with a molecular weight of approximately 23 kDa have a carboxy-terminal extension involved in the inhibition of a second family of CysProt, the C13 legumains [5,6]. In addition, several 85–87 kDa multicystatins, with eight cystatin domains, have been described in dicots [7].

**Figure 1:**
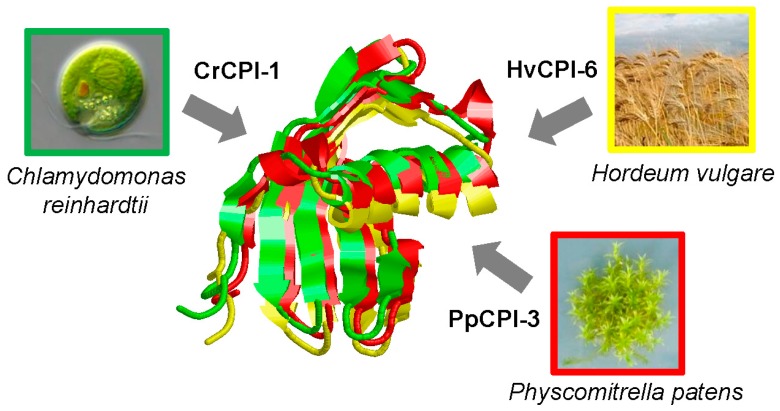
Ribbon plots showing the structural overlay of three-dimensional models for CrCPI-1 (green), PpCPI-3 (red) and HvCPI-6 (yellow) cystatins. Models were created by the SWISS-MODEL program (University of Gen, Belgium) using the known crystal structure of rice OC-I cystatin as template (Protein Data Bank (PDB) identifier 1eqk).

The comparison of the crystal structures of PhyCys from rice, taro, pineapple and sugarcane support a shared globular structure mainly composed by four β-sheets and one α-helix, and without any disulphide bridge [8,9,10,11]. The inhibitory properties of PhyCys are a consequence of a tight and reversible interaction with their target enzymes. It involves a conserved tripartite wedge formed by the partially flexible N-terminus containing one or two glycine residues and two hairpin loops carrying a conserved QxVxG motif and a tryptophan residue, respectively [4]. Minor sequence and structural variations, mainly in hypervariable sites, are implicated in the different target inhibitory potency and specificity among PhyCys [12,13]. Figure 1 displays a structural overlay of PhyCys members from the algae *Chlamydomonas reinhardtii*, the moss *P. patens* and the angiosperm *Hordeum vulgare* that shows the common globular structure and the minor structural variations among PhyCys.

## 2. Phytocystatin Functions

From the functional point of view, PhyCys have been implicated in the regulation of both endogenous and exogenous proteases. CysProt are key enzymes in many physiological processes in plants that have to be tightly controlled. Cystatins have been related to the control of various developmental processes involving CysProt, such as the regulation of protein turnover in storage organs [14,15,16] or the senescence process mediated by abiotic stresses [17,18]. Furthermore, a defence role against pathogens and pests has been inferred to PhyCys from their up-regulation in response to biotic stress-related signals [4]. First reports showed the induction of PhyCys in tomato and soybean by wounding or methyl jasmonate treatments [19,20]. Later on, some publications reported the induction of PhyCys in chestnut, maize and wheat mediated by fungal infection [21,22,23]. Besides, different experimental approaches demonstrated the cystatin induction by insects and acari infestation. Two maize cystatins were identified as induced genes upon attack by *Spodoptera littoralis* caterpillars [24]. Microarray analysis of tomato responses to the spider mite *Tetranychus urticae* feeding revealed the up-regulation of a multicystatin as a defence protein [25]. The silkworm *Bombyx mori* induced the expression of five mulberry cystatin genes, being one of them stable to silkworm gut proteases [26]. Other lines of evidence have corroborated this putative defence role. PhyCys may confer resistance to phytopathogenic virus by inhibiting the cysteine protease activity required to virus replication [27]. Recombinant PhyCys are able to affect the in vitro growth of several phytopathogenic fungi [28,29] using a mechanism that does not involve CysProt activity inhibition [30]. In addition, recombinant PhyCys are able to inhibit the activity of digestive proteases from many herbivores, and a deleterious effect on their development and reproduction occur when herbivores feed in artificial diets including the recombinant PhyCys or in transgenic plants overexpressing a PhyCys gene [4]. Herbivore challenges have been appointed as a key feature in the diversification and proliferation of PhyCys and other protease inhibitors [2,31,32]. This assumption, together with the huge amount of evidence relating PhyCys to herbivore defence leads us to focus this review on the defensive role of PhyCys against phytophagous insects and mites.

## 3. Phytocystatin Targets: Arthropod Proteases

**Figure 2:**
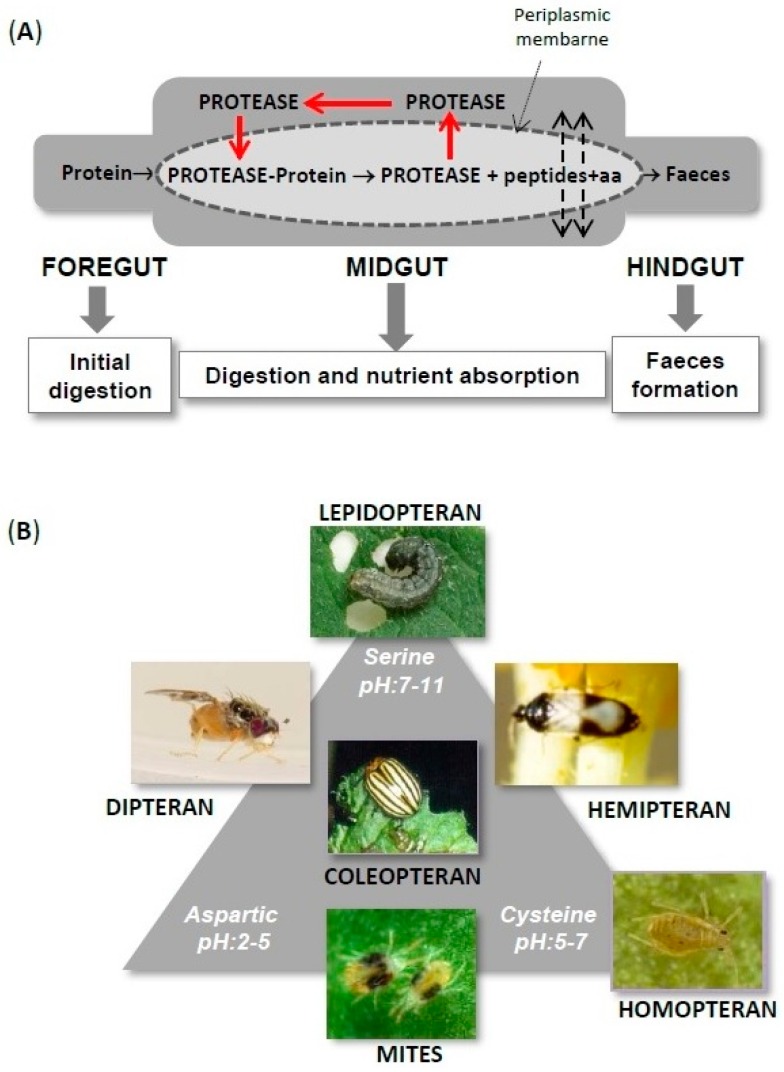
Scheme of arthropod digestive system and protease classes depending of gut pH. (**A**) Digestive system is divided in foregut (initial digestion in some insects), midgut (digestion and nutrient absorption) and hindgut (ion and water regulation and waste excretion), where proteins are degraded by proteases into peptides and amino acids (solid arrows). Degradation products may cross the periplasmic membrane (dotted arrows). Enzyme recycling occurs in the midgut of insects (red arrows) preventing the excretion of digestive proteases; (**B**) Phytophagous arthropods classified depending on their major protease classes (serine-, aspartyl- cysteine-proteases) and the gut pH.

Insect and acari obtain essential nutrients through hydrolytic activities during the digestion process. Thus, an efficient proteolysis of plant proteins is crucial to generate free amino acids for their survival. In fact, because many plant tissues possess suboptimal protein content, nitrogen often becomes the limiting factor in the nutrition of many, if not most, phytophagous arthropods. Since digestive proteases are responsible to catalyse the protein breakdown, these enzymes become potential targets for the control of agricultural pests. Genes encoding proteases are abundantly expressed in gut tissues under a regulatory control during the different developmental stages. Different phytophagous arthropods use different proteases for the digestion process depending on their gut pH. This protease specificity may help to design specific approaches to combat pest using Protease Inhibitors (PIs) (Figure 2).

CysProt that catalyse the hydrolysis of dietary proteins have been characterized in the digestive tract of several groups of phytophagous arthropods, including coleopterans, hemipterans, homopterans and mites (Figure 2B). The majority of plant-feeding coleopterans and hemipterans have slightly acidic midguts and cysteine and aspartyl proteases provide the major midgut proteolytic activities [33]. Serine proteases may also participate in digestion in these two groups, being usually differentially located than CysProt in the gut in coleopterans [34] and in the salivary glands of hemipterans [35]. The digestive proteolytic profile of phloem feeding homopterans [36,37] and cell-content feeding tetranychid mites [38,39] relies as well on CysProt, though aspartyl proteases can be present. CysProt have also been reported in the gut transcripts of herbivorous dipteran [40] and lepidopteran species [41]. Their activities in the guts have only been documented in a few cases [42] and they are expressed at negligible levels [43], being as their physiological role is probably not related to the hydrolysis of dietary proteins. Indeed, non-digestive CysProt are known to be involved in other fundamental functions in arthropods, such as the lysosomal catabolism and processing of proteins, development, reproduction and immune responses [44]. Based on investigations with a number of insects, an ecto-endoperitrophic flow model has been proposed [33] that allows enzyme recycling creating a decreasing gradient of proteases along the midgut and preventing the excretion of enzymes (Figure 2A). In contrast, mite digestive proteases are excreted in the faecal pellets [39]. Genome-wide analysis has revealed multigene families of C1A CysProt genes (both cathepsin B- and L-like) potentially involved in digestion in insects and mites [45]. The hydrolysis of specific substrates, activation by sulfhydryl agents and inhibition by E-64 are usually indicative of the presence of cathepsin B- and L-like proteases in the gut. However, only cathepsin L-like enzymes have been purified to homogeneity from insect midgut contents [46,47,48], suggesting that they may be quantitatively the most important [33]. Likewise, the proteomic analysis of mite faeces from the spider mite *Tetranychus urticae* detected four different cathepsin-L like enzymes, indicating that they represent the most abundant proteases in the gut lumen that are ultimately incorporated into the mite faecal pellets [39]. A unique proliferation of *C13 CysProt* genes (legumain-like) was also identified in the *T. urticae* genome [49] proteolytically active in both mite bodies and faeces [39]. Their functional role in digestion has yet to be elucidated.

Expression levels and activity profiles of CysProt in phytophagous arthropods vary through development and depending on the type of plant consumed. Quantitative changes in activity were reported when comparing larvae and adults of the beetle *Leptinotarsa decemlineata* [50] and when larvae were feed on different plant hosts [51]. Midgut extracts from larvae that ingested eggplant leaves contained only a few protease forms, while numerous forms were observed in extracts of potato-and tomato-fed larvae, being some of these forms specific for a particular diet. Likewise, *T. urticae* mites reared on maize showed significantly higher cathepsin B-like activity than when reared on beans [39]. Transcriptome analysis showed that both C1A and C13 CysProt showed specific developmental patterns of expression [45] and that mites modulate their expression when feeding on different host plants [49]. The reported diet-related changes in digestive enzymes can compensate for variable dietary protein quality and/or quantity, but it may be also a strategy to counteract the effect of plant defence proteins synthesized by different host plants (see Section 5). Mechanisms controlling digestive proteases act at the level of synthesis, secretion and zymogen activation [33]. The process is mediated through the coordinated action of endocrine regulatory peptides, endogenous inhibitors and non-coding micro RNA [52]. Secretion of digestive enzymes is achieved via secretory vesicles that fuse with the apical membrane of the secretory midgut cells. Then, they empty their contents into the gut lumen without any loss of cytoplasm (merocrine), or with the whole (holocrine) or part (apocrine) loss of the secretory cell [53]. In continuously feeding insects the secretion of vesicles is constitutive, whereas in intermittent feeders the secretory vesicles are stored and released into the midgut lumen after feeding. Once delivered, cathepsin L- and B-like CysProt require an enzymatic or autocatalytic activation since they are synthesized as inactive zymogens (pre-pro-proteins encoding a putative signal that targets them for secretion and an autoinhibitory pro-region). The active C1A CysProt is the enzymatic form that can be controlled by endogenous and exogenous cystatins, and maybe also the target for exogenous pro-region sequences [39].

## 4. Tailoring and Selection of Phytocystatin for Pest Control

Cystatins, like other proteins involved in plant-pest interactions, include conserved amino acids affecting their biological activity. These residues have been subjected to a positive selection during the course of their evolution to generate cystatin variants with improved inhibitory potency and specificity towards herbivorous [30,31]. A comparative analysis of cereal cystatins identified highly preserved sequences common to all the cystatins, although some variations maintain the functional diversity within the protein family. The changes are the result of coevolutionary process lead to counterbalance mutations in their pest cognate proteases [54].

In 1998, Koiwa et al. [55] described two soybean cystatins, soyacystatin N and L with 70% sequence identity that showed different inhibition rates. While soyacystatin N was a much more potent inhibitor of *C. maculatus* gut proteases and substantially delayed its growth and development in feeding-diet bioassays, soyacystatin L was essentially inactive as an insecticide. Soyacystatin N variants containing mutations in essential motifs were constructed. Kinetic inhibitory analysis of mutated variants allowed the identification of a novel soyacystatin N isoform with higher affinity to C1A proteases than the wild type [56]. These observations supported the potential of site-directed mutagenesis for the engineering of recombinant PhyCys with improved inhibitory potency toward target arthropods [31,56]. Likewise, tailoring the inhibitory specificity of phytocystatins toward digestive target proteases by single mutations at the positively selected amino acid sites might help to improve their inhibitory function [32].

Kiggundu et al. [31] identified several amino acid sites to be positively selected in cystatins from Poaceae and Solanaceae plants to modulate the inhibitory profile. These sites were located at strategic positions on the protein: surrounding the conserved glycine residues in the N-terminal region, within the first and second inhibitory loops entering the active site of target enzymes, and adjacent to the conserved LARFAV motif in the α-helix. They generated mutants at the 8th cystatin unit of the tomato multicystatin SlCYS8, with alternative residues at positively selected sites. As consequence, these mutants exhibited improved potency against different model CysProt like papain and cathepsins B- and H-like. Furthermore, several variants strongly influenced the inhibitory effectiveness of SlCYS8 against digestive gut CysProt in the Colorado potato beetle [32].

The binding affinity method used to select PhyCys variants with greater insecticidal activity was already described by Koiwa et al. in 1998 [55]. For the same purpose, the discrimination of differentially inhibited *L. decemlineata* CysProt was assessed by biotinylated forms of SlCYS8 and SlCYS8 variants [57]. These tailored cystatins were used to capture susceptible CysProt in protein extracts of the insect midgut by biotin immobilization on avidin-embedded beads. A quantitative LC-MS/MS analysis of the captured proteins was performed to compare the inhibitory profiles within SlCYS8 variants and to identify candidates for the inactivation of specific CysProt targets in the pest [57]. However, rebalancing of midgut proteases by the pest in response to the cystatins may occur. This was observed in *L. decemlineata* larvae, which showed a differential readjustment of functional protease families after feeding single variants of tomato multicystatin SlCYS8 mutated at positively selected amino acid sites [58].

The potato multicystatin PMC is another cystatin composed of eight repeating units, each one capable of inhibiting CysProt [7]. Biochemical studies along with site-directed mutagenesis confirmed the critical role of pH and N-terminal residues in the dynamic transitions between monomer/polymer of PMC. These data support the notion that not only positively selected amino acid sites but also the pH-dependent regulation of the cystatin structure has defence-related implications [7]. Recently, Rasoolizadeh et al. [12] have also confirmed the contribution of closely located amino acids to the functional diversity of positively selected plant cystatins in a broader structure/function context, imposing physicochemical constraints to primary structure alterations.

In barley, several punctual mutants of the HvCPI-1 cystatin were generated and their inhibitory properties against C1A proteases investigated [30]. The variant HvCPI-1 C^68^→G, mutation adjacent to the conserved QxVxG motif, was better inhibitor for papain and cathepsin B-like activities than the wild type. The HvCPI-1 and its C^68^→G variant were tested in vitro against the midgut protease activities from *L. decemlineata.* In consequence, the mutant was selected for potato transformation [59]. A decrease in growth was observed in larvae after feeding on transgenic potato plants expressing the HvCPI-1 C^68^→G cystatin, supporting the improved properties of C^68^→G substitution against the CysProt of this pest.

All these studies underline the complexity of protease/inhibitor interactions in plant-arthropod systems and point out the potential of positively selected amino acids as target sites to tailoring the inhibitory specificity of the cystatins towards CysProt of agronomic significance in crop protection.

## 5. Arthropod Herbivore Responses and Adaptations

Plant-herbivore coevolution has demonstrated that both partners have developed sophisticated mechanisms to overcome defences elaborated by each other.

The impact of dietary PIs in the arthropod depends on their inhibitor properties but also on the arthropod species, on its developmental stage and even it varies within different strains from the same species. Girard et al. [60] described the differential susceptibility of two strains of the cabbage seed weevil *Ceutorhynchus assimilis* to oilseed rape plants overexpressing OC-I cystatin. Zhu-Salzman et al. [61] demonstrated that the growth retardation of *Callosobruchus maculatus* due to soya cystatin was only observed in its earlier stages of the development.

Phytophagous pests rapidly adjust their battery of digestive enzymes in response to the accumulation of defence compounds, mainly to PIs. Comparisons of gut proteolytic activities in the presence or absence of specific PIs have revealed multiple adaptation strategies to circumvent anti-nutritional effects of diet [61,62]. Among them, the overproduction of existing digestive proteases [59,63,64], the secretion of inhibitor resistant enzymes [65,66] and the proteolysis of inhibitors by inhibitor-insensitive digestive enzymes [66,67,68] have been reported as physiological adaptations to minimize the adverse effects on food digestion and nutrient uptake. In some cases, the nutritional stress is compensated by a higher consumption of transgenic tissue than plant control tissue, with the consequent increase in the insect weight [59,63,66]. However, biochemical and molecular approaches evidence that phytophagous arthropods usually combine several adaptive strategies to circumvent PIs [61,69].

Regarding PhyCys, there are some examples describing different physiological mechanisms adopted by insects and acari to avoid the deleterious effects provoked by these PIs. Feeding bioassays conducted with *L. decemlineata* larvae on OC-I-transgenic potato leaves resulted in larger foliage consuming due to the production of OC-I-insensitive proteases as a compensatory response to the nutritional stress [66,70]. In contrast, reduction in weight of *L. decemlineata* larvae fed on potato plants expressing the HvCPI-1 C^68^→G cystatin from barley was probably the result of the metabolic cost associated with the hyperproduction of digestive cathepsin B-like proteases [59]. Zhu-Salzman et al. [61] demonstrated that the cowpea bruchid *C. maculatus* overcame the growth retardation in its earlier developmental stages caused by dietary soya cystatin by increasing the total proteolytic activity and an enzymatic profile shift toward cystatin-insensitive proteases. Consequently, larvae recovered their feeding habits and growth, even in the presence of the cystatin in the diet. Similarly, the spider mite *T. urticae* responded to the ingestion of the barley HvCPI-6 cystatin by increasing the expression of both inhibitor-target and non-target proteases [71]. By using a combination of in vitro protease assays and a shotgun proteomic analysis, Vorster et al. [58] demonstrated that a specific selection of digestive CysProt isoforms is produced in *L. decemlianeata* larvae in response to feeding on single mutated cystatin variants. Curiously, predatory insects can also adapt their digestive metabolism to the presence of PhyCys ingested by their herbivorous preys. Cystatins from rice and barley independently expressed in transgenic potato plants induced digestive compensation in the natural predators *Perillus bioculatus* and *Podisus maculiventris*, respectively, via their herbivorous prey feeding on the plant [59,72,73].

The ingestion of a PI not only induces changes on the pest digestive proteases but also on the global gut content proteome. Proteomic data of the intestinal tract of the coleopteran *C. maculatus* larvae reared with a diet enriched in a cystatin revealed a substantial rearrangement in its proteome [74]. These findings indicate that the digestive adaptation to PIs is part of the arthropod counter-defence but little is still known about the specific molecular mechanisms in the adaptation processes. A wider understanding is needed to use PIs, including PhyCys, for plant permanent protection to pests.

## 6. Biotechnological Cystatin Applications to Control Pests

Some biotechnological strategies have been developed to analyse the effect of a known molecule with insecticide/acaricide properties on phytophagous arthropods. One of them is the production and purification of the molecule of interest as a recombinant product in heterologous systems. The alternative approach is the plant transgenesis through the integration of a desirable gene in the genome of a crop species to confer resistance to a specific pest.

### 6.1. Recombinant Phytocystatins for in Vitro and in Vivo Assays

Recombinant cystatin production using biological systems such as bacteria, yeast or plant cultures, either to perform in vitro inhibitory assays, to be added into artificial diets or even to be sprayed as defence compounds, constitutes an important biotechnological approach. The heterologous expression of PhyCys fused to specific tags to be easily purified from microbial and yeast cultures, or their transient expression in *Nicotiana benthamiana*, allows the isolation of the recombinant protein for further studies or applications [37,75]. Mostly, recombinant cystatins have been used to perform in vitro inhibitory assays using specific protease substrates and protein extracts or purified digestive proteases from arthropods. This approach generates rapid and useful information about the proteolytic profiles and cystatin binding affinities [37,38,76,77] and is particularly used for the tailoring and selection of PhyCys for pest control (see Section 4).

Another application of recombinant cystatins is based on the fact that some insects, at least certain laboratory strains, may feed on artificial diets. Thus, feeding bioassays can be performed by adding recombinant cystatins to diets to evaluate insect susceptibility. Many reports have tested differences in insect growth, development and survival as well as alterations in digestive physiology in response to a cystatin-artificial diet under confined conditions (Koiwa et al. 2000 [37,46,78]).

**Figure 3:**
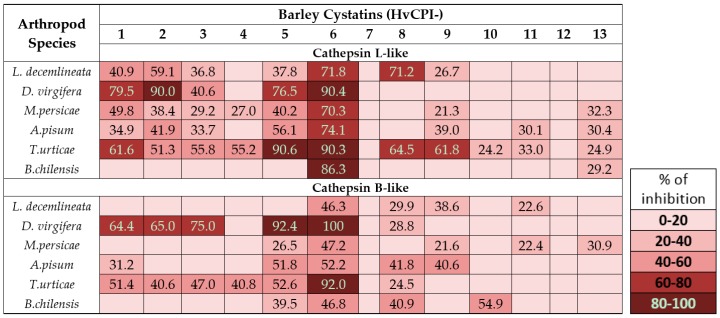
In vitro inhibitory activity of the recombinant barley cystatins (HvCPI-1 to HvCPI-13) against cathepsin L- and B-like activities in several phytophagous arthropods.

In barley, the inhibitory capability of the whole gene family of cystatins, containing 13 members, was analysed (see Suplementary materials) after purifying the 13 recombinant cystatins (HvCPI-1 to -13). First, in vitro inhibitory experiments were performed against protein extracts from two aphids, *M. persicae* and *Acyrthosiphon pisum*, and two mites, *T. urticae* and *Brevipalpus chilensis*, which rely on CysProt for digestion [37,38]. Now, to complete this study, the in vitro assays have been extended by including two coleopteran species, *L. decemlineata* and *Diabrotica virgifera*, whose major digestive proteases also belong to the C1A peptidase family [33]. Protein extracts from the coleopteran were prepared to carry out in vitro inhibitory assays with the 13 recombinant barley cystatins, using two substrates susceptible to be specifically degraded by cathepsin B- and L-like proteases. As is shown in Figure 3, protease activities from *D. virgifera* resulted more susceptible to be inhibited by barley cystatins than the enzymes from Colorado potato beetle. The inhibitory profile indicated that cathepsin L-like activity of *L. decemlineata* was high inhibited by barley cystatins than the B-like. Both activities were similarly reduced in the case of the western corn rootworm. Recombinant HvCPI-6 was the strongest inhibitor and reduced about 70% and 50% cathepsin L- and B-like activities in *L. decemlineata*, respectively, and almost produced a complete inhibition of both activities in *D. virgifera* extracts. The cystatins HvCPI-1, -2, -3 and -5 also inhibited both cathepsin activities in extracts from both coleopterans whereas HvCPI-8 only reduced the cathepsin L-like activity of Colorado potato beetle. No significant inhibition was detected when HvCPI-4, -7, -10, -12 and -13 proteins were used (Figure 3).

The whole set of inhibitory results obtained with the recombinant barley cystatins and the protein extracts from pest species demonstrated an inhibitory specificity of the different PhyCys against C1A proteases from different arthropod origin. This specificity is essential to select the best inhibitor for controlling each arthropod species, or even a particular strain, in an integrated pest management program. Our findings also indicate that the HvCPI-6 cystatin resulted especially efficient, which has been confirmed using artificial diets [37] and after being expressed in transgenic plants (see Section 6.2).

### 6.2. Phytocystatins and Transgenic Plants

The experience of more than twenty years of commercialization of Bt-crops expressing *Bacillus thuringiensis* toxins to control insect pests and reduce insecticide spray practises supports the usefulness of crop biotechnology [79]. Nevertheless, some phytophagous arthropods, mainly aphids and mites, are insensitive to Bt proteins [80,81]. For instance, the two-spotted spider mite *T. urticae* fed on Bt-plants is able to accumulate higher levels of Cry toxins than that expressed by transgenic plants without suffering any alteration in their behaviour [82]. Similarly, no differences have been reported in the performance of the green peach aphid, *M. persicae* that fed on Bt or non-Bt plants, although Bt toxin residues have not been found in their guts [83]. In this scenario, PIs appeared as alternative genes against the digestive physiology of insects and mites.

There are many examples of transgenic plants expressing cystatin encoding genes as well as engineered cystatin genes with improved potency or specificity to control pests (Table 1). Numerous transgenic plants harbouring cystatin from rice, barley, *Arabidopsis*, potato and taro have been generated with the aim of controlling insects and acari whose digestive physiology is mainly based on CysProt. As is shown in Table 1 and Table 2, rice cystatin encoding genes, particularly OC-I, OC-II and the engineered OC-IΔ86 form, are the most widely used cystatin as transgenes. Two cystatins from barley (HvCPI-1 and HvCPI-6) and the point mutant HvCPI-1 C^68^→G have been transgenically expressed in *Arabidopsis*, potato and maize to determine how they affect insects and mites. In addition, *Arabidopsis* and taro cystatins and potato multicystatin have been ectopically expressed in poplar and solanaceous species to analyse their inhibitory properties against insects.

From the insect side, coleopteran species are probably the most frequently employed pests to perform bioassays with cystatin-transgenic plants. Among them, many studies highlighted changes in the digestive physiology, performance and adaptive effects of the Colorado potato beetle *T. decemlineata* after feeding on potato plants independently expressing OC-I, OC-II and HvCPI-1 cystatin [59,66,70,72,73,84,85] or combining more than one PI [62,86,87]. The homopteran *M. persicae* has been also used to perform bioassays with potato, oilseed rape and eggplant expressing the OC-I or its mutated form OC-IΔ86 [88,89,90,91,92]. These studies revealed that rice cystatins reduced proteolytic activities of the aphid and interfere with various aspects of its performance such as weight or fecundity. Likewise, significant delay in the development time to reach adult stage was observed in *M. persicae* after feeding *Arabidopsis* expressing HvCPI-6 [37]. In addition, feeding assays to check the cystatin impact on hemipteran and lepidopteran species using transformed solanaceous and fabaceous species have demonstrated the defence role of this PI alone or in combination with other transgenes, even when CysProt are not the major digestive proteases of the pest (Table 1 and Table 2).

To avoid pest adaptation to PI-plants, the gene pyramiding approach by stacking various defence genes in a transgenic plant has been developed as an excellent biotechnological method. Besides, the creation of hybrid PIs by combining PI genes or derived partial sequences may enhance their activity or increase the potential number of interactions in the target pest [13]. Plastid engineering technology combined with transgene stacking approach has provided the generation of tobacco multi-resistance against insects and pathogens. This strategy confirmed that the simultaneous expression of several defence genes conferred a broad spectrum of resistance [93]. A special mention is required to highlight the potential of engineered multidomain cystatins, consisting in multiple domains from different cystatins in a single protein. Using this technology, Outchkourov et al. [94,95] combined deterrent with toxic effects derived from an engineered inhibitor expressed in transgenic potatoes. As a result, they provided a new control way to effectively interfere with growth and development of the western flower thrip *Frankiniella occidentalis*.

The importance of PhyCys to combat phytophagous tetranychid mites is remarkable. Few approaches have been useful to control the two-spotted spider mite *T. urticae*, an important polyphagous pest that feeds on more than 150 crop species and presents a great record of pesticide [105]. However, feeding experiments developed with transgenic maize or *Arabidopsis* resistant plants expressing HvCPI-6 cystatin showed a significant reduction of the mite cathepsin L- and B-like activities. In parallel, the development and reproductiveperformance of mites was negatively affected [38,71]. The multigene approach targeted to control *T. urticae* infestation by co-expressing two barley proteases inhibitors (the HvCPI-6 cystatin and the trypsin inhibitor CMe) in *Arabidopsis* plants corroborated the impact of the inhibitors on the endogenous mite peptidase activities and on the mite survival. Interestingly, double transgenic plants showed significantly less damaged leaf area than plants from single transformation events and much less than non-transformed controls [71].

The widespread use of transgenic plants with cystatin alone, stacked with other genes, or with engineered cystatin specifically targeting pest digestive proteases has demonstrated its potential for pest control.

## 7. Conclusions and Future

Many advances have been achieved on the characterization and function of PhyCys in different physiological processes of the plant involving the regulation of endogenous or heterologous proteases. Their defensive function has pushed researchers to consider PhyCys as proteins of particular value with big potential to be integrated as a new tool in Pest Control Management. Most of the strategies developed up to now have been based on their transgenic expression in plants. Nevertheless, some biochemical characteristics of cystatins, such are their small size or their stability, have redirected the interest for their production as recombinant molecules. The large-scale production of PhyCys has already demonstrated their utility as nutraceuticals, food additives or stabilizers of other recombinant proteins [13]. Probably, the evaluation of their potential as chemicals with insecticide and acaricide properties to be sprayed is the next step. A more complete understanding of the arthropod responses to PI ingestion is a prerequisite for the application of PhyCys in the field.

## Figures and Tables

**Table 1:** Target arthropods and transgenic plants overexpressing phytocystatins (PhyCys).

Target Pest		Cystatin	Transgenic Plant	Reference
Order	Species			
Homoptera	*Myzus persicae*	OC-I	Potato	[88]
			Oilseed rape	[89,90]
			Eggplant	[92]
		OC-I∆D86	Potato	[91]
		HvCPI-6	*Arabidopsis*	[37]
Coleoptera	*Baris coerulescens*	OC-I	Oilseed rape	[96]
	*Ceutorhynchus assimilis*	OC-I	Oilseed rape	[60,63]
	*Chrysomela tremulae*	OC-I	Poplar	[97]
	*Chrysomela populi*	AtCYS	Poplar	[98]
	*Leptinotarsa decemlineata*	OC-I	Potato	[66,72,73,74,84]
		OC-II	Potato	[85]
		HvCPI-1 C→G	Potato	[59]
	*Phytodecta fornicata*	OC-II	Alfalfa	[99]
	*Psylliodes chrysocephala*	OC-I	Oilseed rape	[63]
	*Sitophilus zeamais*	OC-I	Rice	[100]
Lepidoptera	*Plutella xylostella*	OC-I	Oilseed rape	[101]
	*Spodoptera littoralis*	HvCPI-1 C→G	Potato	[38]
Hemiptera	*Macrosiphum euphorbiae*	OC-I	Eggplant	[92]
		OC-I∆D86	Potato	[91]
	*Riptortus clavatus*	CCI	Soybean	[102]
Acarina	*Brevipalpuls chilensis*	HvCPI-1 C→G	Potato	[38]
	*Tetranychus urticae*	HvCPI-6	Maize	[38]
		HvCPI-6	*Arabidopsis*	[39,71]

Cystatins: *Arabidopsis* (AtCYS), Barley (HvCPI-1 C→G, HvCPI-6), Maize (CC-I) and Rice (OC-I, OC-IΔD86, OC-II).

**Table 2:** Target arthropods and gene pyramiding or engineered approaches using PhyCys.

Target Pest		Cystatin + Proteins	Transgenic Plant	Reference
Order	Species			
Coleoptera	*Leptinotarsa decemlineata*	OCI + OCII	Potato	[86,87]
		OC-I∆D86 + CpTI	*Arabidopsis*	[62]
	*Plagiodera versicolora*	OC-I + CRY3A	Poplar	[103]
Lepidoptera	*Helicoverpa armigera*	CeCPI + Sporamin	Tobacco	[104]
	*Spodoptera exigua*	CeCPI + Sporamin + chitinase	Tobacco	[93]
	*Spodoptera littura*	CeCPI + Sporamin + chitinase	Tobacco	[93]
Thysanoptera	*Frankiniella occidentalis*	Engineered PCM + domains	Potato	[95]
Acarina	*Tetranychus urticae*	HvCPI-6 + CMe	*Arabidopsis*	[70]

Cystatins: Barley (HvCPI-6), Rice (OC-I, OC-IΔD86, OC-II), Taro (CeCPI), Potato (PCM). Trypsin Protease Inhibitors: Cowpea (CpTI), Barley (CMe). Bt-Toxin (CRY3A).

## Supplementary Materials

Supplementary materials can be found at www.mdpi.com/1422-0067/17/10/1747/s1.
