# Supplementary material for: Phytocystatins: Defense Proteins against Phytophagous Insects and Acari
Source: Int J Mol Sci. 2016 Oct 20;17(10):1747. doi: 10.3390/ijms17101747 (PMC5085774; doi:10.3390/ijms17101747)
Supplement: Supplementary file 1 [file ijms-17-01747-s001.pdf]

Supplementary Materials: Phytocystatins: Defense Proteins against Phytophagous Insects and Acari

Manuel Martinez, Maria Estrella Santamaria, Mercedes Diaz-Mendoza, Ana Arnaiz, Laura Carrillo, Felix Ortego and Isabel Diaz

Material and Methods

Inhibitory Activities of Barley Cystatins against L. decemlineata and Diabrotica virgifera

Guts from adults of Colorado potato beetle, *L. decemlineata* and from larvae of the western corn rootworm *D. virgifera* were dissected, subsequently homogenized in 0.15 M NaCl and centrifuged at 10,000× *g* for 5 min. Supernatants were pooled and total protein content was determined according to the Bradford method (1976) [1]. Inhibitory activity of the thirteen barley cystatins (HvCPI-1 to -13) was in vitro tested. Basically, 1 µg and 7.5 µg of gut protein extracts from *L. decemlineata* and *D. virgifera*, respectively, were preincubated for 10 min with 0.4 µg of each cystatin in a buffer 100 mM sodium phosphate pH 6.0, L-cysteine, 10 mM EDTA, and 0.01% (*v*/*v*) Brij35. Subsequently, Z-FR-AMC and Z-RR-AMC (*N*-carbobenzoxyloxy-Arg-Arg-7-amido-4-methylcoumarin) specific substrates to be degraded by cathepsins L- and B-like were added at a final concentration of 0.2 mM, and incubated at 30 °C. Fluorescence was measured using an excitation filter of 365 nm and an emission filter of 465 nm (Tecan GeniusPro, Männedorf, Switzerland). The system was calibrated with known amounts of AMC hydrolysis product in a standard reaction mixture. Results were expressed as a percentage of protease activity relative to that in the absence of the cystatin inhibitor. All assays were carried out in triplicate and blanks were used to account for spontaneous breakdown of substrates.

Reference

1. Bradford, M.M. A rapid and sensitive method for the quantification of microgram quantities of protein utilizing the principle of protein-dye binding. *Anal. Biochem.* **1976**, *72*, 248–254.
